# Three amino acid residues are required for the recognition of *Ralstonia solanacearum* RipTPS in *Nicotiana tabacum*

**DOI:** 10.3389/fpls.2022.1040826

**Published:** 2022-10-13

**Authors:** Yuyan An, Jialan Chen, Zhangyan Xu, Xue Ouyang, Peng Cao, Rongbo Wang, Peiqing Liu, Meixiang Zhang

**Affiliations:** ^1^ National Engineering Laboratory for Endangered Medicinal Resource Development in Northwest China, Key Laboratory of Medicinal Resources and Natural Pharmaceutical Chemistry of Ministry of Education, College of Life Sciences, Shaanxi Normal University, Xi’an, China; ^2^ Department of Plant Pathology, Nanjing Agricultural University, Nanjing, China; ^3^ Fujian Key Laboratory for Monitoring and Integrated Management of Crop Pests, Institute of Plant Protection, Fujian Academy of Agricultural Sciences, Fuzhou, China

**Keywords:** RipTPS, avirulence, virulence, *Ralstonia solanacearum*, plant immunity

## Abstract

*Ralstonia solanacearum* causes devastating diseases in a wide range of economically important crops. It secretes a large number of virulence factors, also known as effectors, to promote its infection, and some of them are recognized when the host plant contains corresponding resistance genes. In this study we showed that a type III effector RipTPS from the avirulent *R. solanacearum* strain GMI1000 (RipTPS_G_) specifically induced cell death in *Nicotiana tabacum*, but not in *Nicotiana benthamiana*, whereas the RipTPS homolog in the virulent strain CQPS-1 (RipTPS_C_) induced cell death in neither *N. tabacum* nor *N. benthamiana*. These results indicated that RipTPS_G_ is recognized in *N. tabacum*. Expression of RipTPS_G_ induced upregulation of hypersensitive response (HR) -related genes in *N. tabacum*. The virulence of CQPS-1 was reduced when RipTPS_G_ was genetically introduced into CQPS-1, further confirming that RipTPS_G_ functions as an avirulence determinant. Protein sequence alignment indicated that there are only three amino acid polymorphisms between RipTPS_G_ and RipTPS_C_. Site-directed mutagenesis analyses confirmed that the three amino acid residues are jointly required for the recognition of RipTPS_G_ in *N. tabacum*. Expression of either RipTPS_G_ or RipTPS_C_ suppressed flg22-triggered reactive oxygen species (ROS) burst in *N. benthamiana*, suggesting that RipTPS contributes to pathogen virulence. Mutating the conserved residues in RipTPS’s trehalose-phosphate synthase (TPS) domain did not block its HR induction and defense suppression activity, indicating that the TPS activity is not required for RipTPS’s avirulence and virulence function.

## Introduction

The bacterial pathogen *Ralstonia solanacearum* is a destructive phytopathogen that attacks many plants over a broad geographical range (Genin and Denny, 2012). It is a soil-borne bacterium which can infect more than 200 plant species, including many important crops such as potato, tobacco, tomato, and some ornamental plants (Niu et al., 2022). The extensive genetic diversity of strains causes various bacterial wilt diseases, leading to great economic losses worldwide every year (Qi et al., 2022). In-depth study of the molecular mechanism underlying the interaction between *R. solanacearum* and plants has important theoretical significance for formulating new disease control strategies.

Plant immunity relies on two levels of pathogen perception that trigger defense mechanisms, pattern-triggered immunity (PTI) and effector-triggered immunity (ETI) (Jones and Dangl, 2006). PTI is the first layer of plant immunity, which involves the recognition of conserved microbial elicitors termed pathogen-associated molecular patterns (PAMPs), such as flagellin, cold shock protein, and elongation factor Tu, by specific plasma membrane receptors (Jones and Dangl, 2006). The receptors then activate signaling cascades in the host cell to restrict pathogen growth (Segonzac et al., 2011). Reactive oxygen species (ROS) burst is a hallmark event of PTI signaling (Segonzac et al., 2011). For example, flg22, a conserved 22-amino-acid peptide from bacterial flagellin, is a PAMP which often triggers ROS burst in host plants (Nakano and Mukaihara, 2019b; Mittler et al., 2022). However, to overcome host PTI for successful infection, adapted pathogens have evolved the ability to inject effectors inside the host cell. In turn, to counter effector-mediated suppression of PTI, plant further evolves a second layer of immunity, ETI, which is based on internal recognition of effector proteins by cytoplasmic receptors. Compared with PTI, ETI initiates stronger and more prolonged defense responses (Jones and Dangl, 2006), which often results in programmed cell death called hypersensitive response (HR) to restrict bacterial multiplication at the infection site (Mukaihara et al., 2016). Although PTI and ETI are initiated by distinct activation mechanisms, they share similar contents of defense responses (Tsuda and Katagiri, 2010). PTI and ETI are mutually linked, and potentiate each other (Ngou et al., 2021; Yuan et al., 2021).

As one of the most destructive plant pathogens, *R. solanacearum* has gained increasing attention. Genome sequencing of several representative strains of *R. solanacearum*, such as GMI1000, has broadened our knowledge of the evolution and speciation of this pathogen and led to the increasing identification of molecular determinants involved in pathogenicity and host-range specificity. *R. solanacearum* employs a type III secretion system (T3SS) which secretes more than 70 type III effectors (T3Es) into plant cells to promote infection (Genin and Denny, 2012). These effectors are powerful weapons for bacterial pathogens, but may also lead to potential recognition of the pathogens by plant immune system (Sang et al., 2020). The T3Es recognized by plant resistance proteins to activate ETI are called avirulence determinants. They restrict pathogen virulence to specific plants and often determine the host-range specificity of a pathogen (Nakano and Mukaihara, 2019b).

GMI1000 is the first genome sequenced *R. solanacearum* strain and has been used as a model strain for *R. solanacearum*-related study (Poueymiro et al., 2009). To date, many T3Es of GMI1000 have been studied to analyze their avirulence or virulence functions (Sun et al., 2017; Morel et al., 2018; Sang et al., 2020; Xian et al., 2020; Yu et al., 2020; Cheng et al., 2021; Wang et al., 2021; Niu et al., 2022; Qi et al., 2022), although very few of them have been functionally characterized thoroughly in planta. Several of the reported effectors have been shown to elicit HR and act as avirulence factors in certain plants. For example, PopP1 and PopP2, two members of the YopJ/AvrRxv family, confer avirulence in *Petunia* as well as *Nicotiana gultinosa* (Lavie et al., 2002; Poueymiro et al., 2009) and Arabidopsis (Deslandes et al., 2003), respectively. AvrA is an avirulence determinant recognized by both *Nicotiana tabacum* and *Nicotiana benthamiana* (Poueymiro et al., 2009). RipAX2 triggers specific resistance and hence is specifically recognized by eggplant AG91-25 (Morel et al., 2018). In addition, it was indicated that RipE1 is an avirulence determinant recognized by *N. benthamiana* (Sang et al., 2020) and RipAW is recognized by both *N. benthamiana* and *N. tabacum* (Niu et al., 2022). Except for GMI1000, Rip36 and RipB from another *R. solanacearum* strain RS1000 have also been suggested as avirulent factors in *S. torvum* and *N. benthamiana*, respectively (Nahar et al., 2014; Nakano and Mukaihara, 2019b). Identification of the above virulent and avirulent factors provide extraordinary insights into the interaction between *R. solanacearum* and its host/nonhost plants. However, among the extensive repertoire of T3Es, only a small fraction has been studied in depth. Therefore, there is still a long way for us to unravel the story of the non-stop battle between *R. solanacearum* and plants.

Trehalose-6-Phosphate synthesized by trehalose-phosphate synthase (TPS) is a signaling metabolite and plays important roles in plant growth and flowering regulation (Schluepmann et al., 2012; Wahl et al., 2013). Interestingly, a role for trehalose metabolism is merging in pathogen-plant interaction (Tournu et al., 2013). For example, synthesis of the disaccharide trehalose by *Pseudomonas aeruginosa* strain PA14 is required for pathogenesis in Arabidopsis (Djonovic et al., 2013). In *R. solanacearum* species complex, RipTPS is a conserved T3E which has been reported to endow with a TPS enzymatic activity (Poueymiro et al., 2014). In that report, it was demonstrated that RipTPS could specifically elicit a hypersensitive-like response on *N. tabacum*, suggesting its role as a potential avirulent factor in tobacco. Unexpectedly, the TPS activity was not involved in RipTPS-elicited hypersensitive-like response (Poueymiro et al., 2014). It will be interesting to further characterize the function of RipTPS and the role of its TPS activity.

CQPS-1 is a *R. solanacearum* strain newly isolated from a highland and its genome has been sequenced (Liu et al., 2017). Most genes coding core T3Es were conserved in CQPS-1 compared with the model strain GMI1000. However, CQPS-1 can infect *N. tabacum* but GMI1000 cannot. Here, we found that only three amino acid polymorphisms existed between RipTPS in GMI1000 (RipTPS_G_) and CQPS-1 (RipTPS_C_) strains. The natural variation between these two RipTPS alleles will undoubtedly facilitate the function characterization of RipTPS. Therefore, using these materials, we provided strong evidence for the confirmation of RipTPS_G_ as an avirulence determinant in *N. tabacum*, and proved that the three amino acid polymorphisms jointly determine the recognition of RipTPS_G_ in *N. tabacum*. We also investigated whether RipTPS retained its ability to suppress plant immune responses by analyzing its effect on flg22-triggered ROS burst. Finally, roles of TPS activity in both RipTPS_G_-elicited HR in nonhost *N. tabacum* and RipTPS_G_-inhibited ROS burst induced by flg22 were evaluated through site mutagenesis analyses.

## Materials and methods

### Plant growth conditions and bacterial strains

*Nicotiana benthamiana* and *Nicotiana tabacum* plants were grown at 24°C in a walk-in chamber under long-day conditions (16 h light/8 h dark). *Agrobacterium tumefaciens* strain GV3101 was used to transiently express effectors in tobacco leaves. *Esch*e*richia coli* strain DH5α was used for vector construction. They were cultured on Luria-Bertani (LB) agar plates or in LB liquid medium with proper antibiotics at 28°C and 37°C, respectively. *Ralstonia solanacearum* GMI1000 and CQPS-1 were grown on Bacto-agar and glucose (BG) medium at 28°C.

### Sequence analysis

The sequence alignment was performed using SeqHunter software (Ye et al., 2010).

### *Agrobacterium*-mediated transient expression

The agrobacterial cells with corresponding constructs were suspended in an infiltration buffer containing 150 μM acetosyringone, 10 mM MgCl_2_ and 10 mM MES (pH 5.6), and were incubated at 28°C for 2 hours after being adjusted to an OD_600_ of 0.5. Then the bacterial suspensions were infiltrated into the fully expanded leaves of 5-week-old tobacco with a needless syringe.

### RNA extraction and quantitative PCR

Total RNA was extracted using the RNAsimple Total RNA Extraction Kit (TIANGEN). The RNA sample was then reverse transcribed in a 20-μL volume using the HiScript II Q Select RT SuperMix for qPCR kit (Vazyme). SYBR Green quantitative PCR was performed to determine the relative expression levels of HR-related genes *NtHIN1* and *NtHsr203J*, and *NtEF1α* was selected as an internal control. Quantification of the relative changes in gene transcript levels was performed using the 2^−ΔΔCt^ method (Livak and Schmittgen, 2001). The primers used for amplification were listed in **Table S1**.

### Measurement of ROS burst

The ROS production level was measured as previously described (Segonzac et al., 2011) with slight modifications. Briefly, leaf disks were taken from 5-week-old *N. benthamiana* plants, and then floated overnight in 200 μL sterile distilled water. Before measurement, water was replaced with 100 μL reaction solution containing 17 μg/mL luminol, 10 μg/mL horseradish peroxidase and 100 nM flg22. The ROS production level was monitored using a Glomax-96 Microplate Luminometer (Promega).

### Generation of transgenic CQPS-1 expressing *RipTPS*_G_

*R. solanacearum RipTPS*_G_ was cloned into pHM1 vector with *Eco*RI/*Hind*III. The recombinant plasmid was introduced into CQPS-1 by electroporation.

### Site-directed mutagenesis of RipTPS_G_ and generation of *RipTPS*_G_ knockout mutants

*R. solanacearum RipTPS*_G_ was cloned into pENTR vector. PCR-based site-directed mutagenesis was performed to mutate the aspartic 23 of RipTPS_G_ into glycine, serine 290 into arginine, alanine 483 into valine, tyrosine 154 into valine, tryptophan 163 into serine, and aspartic 208 into glycine, respectively. The LR reaction was conducted to clone RipTPS_G_^D23G^, RipTPS_G_^S290R^, RipTPS_G_^A438V^, RipTPS_G_^Y154V^, RipTPS_G_^W163S^, and RipTPS_G_^D208G^ into pGWB505, respectively. All the primers used for this experiment were listed in **Table S1**.

The upstream and downstream fragments of *RipTPS*_G_ were separately amplified from GMI1000 genomic DNA and fused using overlap PCR, and the resulting fragment was inserted into the *Xba*I/*Bam*HI sites of pK18mobsacB (Schafer et al., 1994). Then the recombinant pK18mobsacB vector was transformed into *R. solanacearum* GMI1000, and the *RipTPS* deletion mutants were generated by homologous recombination-based procedures. Primers used for generation and identification of *RipTPS*_G_ knockout mutants were listed in **Table S1**.

### *R. solanacearum* infection assay

For leaf inoculation*, R. solanacearum* strains were grown in liquid BG medium at 28°C overnight. Bacterial cells were collected by centrifugation and washed with sterile water and adjusted to a final OD_600_ of 0.001. *N. tabacum* leaves were infiltrated with the bacterial solution. The bacterial titers were measured three days after inoculation.

For *R. solanacearum* soil drenching inoculation, 30-day-old *N. tabacum* plants were used. *R. solanacearum* was grown overnight at 28°C in BG liquid medium till OD_600_ to 2.0, then centrifuged and suspended in distilled water. Plants grown in pots were inoculated *via* soil drenching with a bacterial suspension (OD_600_ = 0.1). Scoring of visual disease symptoms on the basis of a scale ranging from ‘0’ (no symptoms) to ‘4’ (complete wilting) was performed as previously described (Vailleau et al., 2007).

### Statistical analysis

All experiments were performed with at least three biological replicates, and data were analyzed using SPSS 20.0 software. Student’s *t*-test was performed along with analysis of variance to compare the differences between treatments.

## Results

### RipTPS_G_ induces HR on *N. tabacum*, but RipTPS_C_ cannot

**Figure 1:**
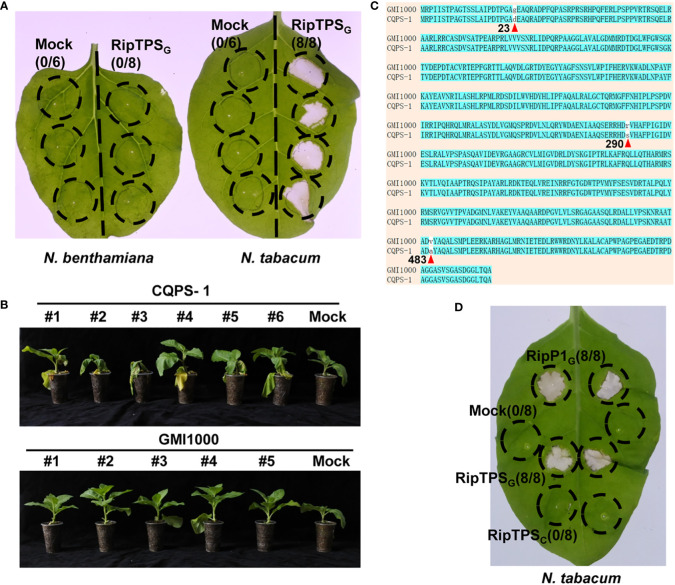
RipTPS_G_ (RipTPS from GMI1000), but not RipTPS_C_ (RipTPS from CQPS-1), elicits hypersensitive response (HR)-like phenotype in *Nicotiana tabacum*. **(A)** RipTPS_G_ elicits an HR-like phenotype in *N. tabacum*, but not in *Nicotiana benthamiana*. **(B)** Phenotypes of *N. tabacum* infected with *R. solanacearum* strain GMI1000 or CQPS-1 11 days after root inoculation. **(C)** Pairwise sequence alignment of RipTPS proteins derived from avirulent GMI1000 and virulent CQPS-1. Positions of the three amino acid polymorphisms are indicated by red triangles and the corresponding numbers below the amino acids. **(D)** RipTPS_G_ elicits an HR-like phenotype in *N. tabacum*, but RipTPS_C_ cannot. For A and D, leaves of *N. benthamiana* or *N. tabacum* were infiltrated with *Agrobacterium tumefaciens* GV3101 carrying LTI6b (Mock, a negative control), RipP1_G_ (RipP1 from GMI1000, a positive control), RipTPS_G_ or RipTPS_C_. Photographs were taken 24 hours after infiltration. Circles indicate the infiltrated area on the leaf panels. The fraction in brackets represents the number of HR over the total number of the infiltrated leaves.

*R. solanacearum* strain GMI1000 cannot infect *N. tabacum*, indicating GMI1000 is recognized in *N. tabacum*. To identify the potential avirulence determinants in GMI1000 that are specifically recognized in *N. tabacum*, we screened thirteen type III effectors from GMI1000 by observing their ability to induce HR in *N. tabacum*. This assay led to the identification of an effector RipTPS, which only induced HR in *N. tabacum*, but not in *N. benthamiana* (**Figure 1A**). This result is consistent with a previous report (Poueymiro et al., 2014), indicating that RipTPS in GMI1000 (RipTPS_G_) may be recognized in *N. tabacum*.

CQPS-1 is another *R. solanacearum* strain whose type III secretion system cluster is conserved compared with GMI1000 (Liu et al., 2017). In contrast to GMI1000, CQPS-1 can infect *N. tabacum* (Liu et al., 2017, **Figure 1B**). Here, pairwise sequence alignment of RipTPS derived from avirulent GMI1000 and virulent CQPS-1 strains showed that there are only three amino acid polymorphisms between them (**Figure 1C**). Interestingly, the RipTPS from CQPS-1 (RipTPS_C_) cannot induce HR in *N. tabacum* (**Figure 1D**), further suggesting that RipTPS_G_ is recognized in *N. tabacum*.

**Figure 2:**
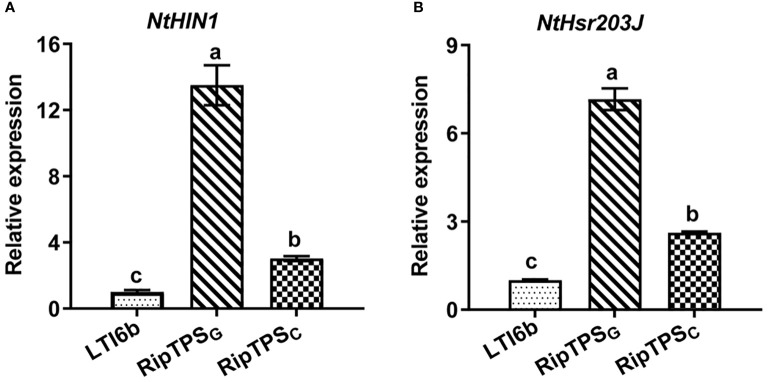
RipTPS_G_ upregulates expression of HR-related genes in *N. tabacum*. Leaves of *N. tabacum* were infiltrated with *A tumefaciens* GV3101 carrying LTI6b (Control), RipTPS_G_ or RipTPS_C_. The expression of *NtHIN1* **(A)** and *NtHsr203J* **(B)** was quantified by qRT-PCR at 19 hpi. Data are means ± standard errors (SE, n = 9). The different letters indicate significant differences at *p* ≤ 0.01.

To determine whether RipTPS_G_ triggers ETI-like response, expression of two HR-related genes *NtHIN1* and *NtHsr203J* was measured. The result showed that RipTPS_G_ dramatically enhanced relative expression of *NtHIN1* and *NtHsr203J* in *N. tabacum* compared with LTI6b control which is a membrane-localized protein unrelated to plant immunity, whereas their induction was significantly compromised in RipTPS_C_ (**Figure 2**). Together, these results indicate that RipTPS_G_ is an avirulence protein recognized in *N. tabacum*.

### Expression of *RipTPS*_G_ in CQPS-1 reduces its virulence on *N*. *tabacum*

**Figure 3:**
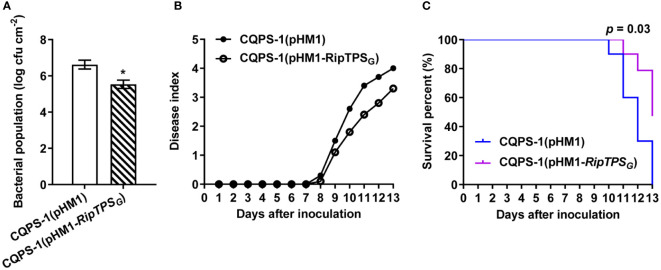
Expression of *RipTPS*_G_ in CQPS-1 reduces pathogen virulence in *N. tabacum.* **(A)** Titers of *R. solanacearum* strain CQPS-1. CQPS-1 cells expressing the empty vector (pHM1) and *RipTPS*_G_ (pHM1-RipTPS_G_) were separately inoculated on 30-day-old *N. tabacum* leaves and bacterial population was quantified 2 days after inoculation. * indicates significant difference at *p* ≤ 0.05. **(B)** Disease index of 30-day-old *N. tabacum* after soil drenching inoculation of CQPS-1(pHM1) or CQPS-1(pHM1-RipTPS_G_). Fifteen plants were observed per genotype per biological replicate. **(C)** Survival analysis of the data in **(B)**. Statistical analysis was performed using a Log-rank (Mantel-Cox) test (n = 45), and the corresponding *p* value is shown in the graph.

To genetically investigate the function of RipTPS_G_ as an avirulence determinant, we expressed *RipTPS_G_* in CQPS-1 and investigated whether it can affect virulence of CQPS-1 on *N*. *tabacum.* Results showed that, compared to the empty vector control, expressing *RipTPS*_G_ significantly decreased CQPS-1’s population in the inoculated *N*. *tabacum* leaves (**Figure 3A**). In addition, expressing *RipTPS*_G_ in CQPS-1 largely reduced the disease index (**Figure 3B**) and consequently enhanced the survival percent (**Figure 3C**) of *N*. *tabacum*. These results indicate that expression of *RipTPS*_G_ significantly reduced virulence of CQPS-1 on *N. tabacum*, confirming RipTPS_G_ as an avirulence determinant in *N. tabacum*.

### The three amino acid residues jointly determine the recognition of RipTPS_G_ in *N*. *tabacum*

**Figure 4:**
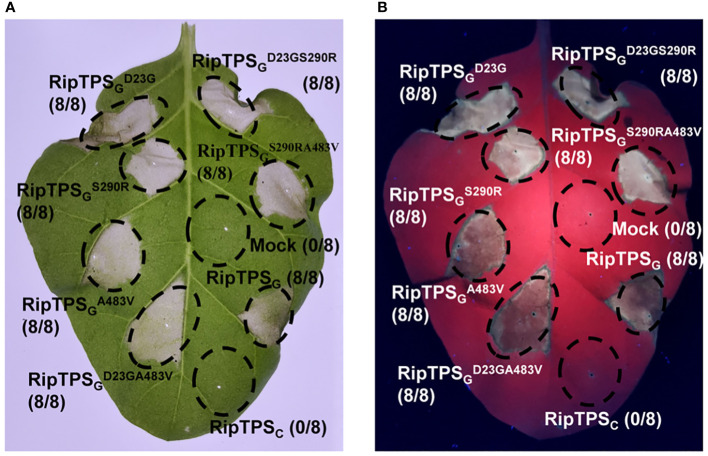
Three amino acid residues are jointly required for RipTPS_G_-induced HR in *N. tabacum*. Leaves of *N. tabacum* were infiltrated with GV3101 carrying LTI6b (Mock, Control), RipTPS_G_, RipTPS_C_, or RipTPS_G_ mutants. Photographs were taken 36 hours after infiltration under white light **(A)** or ultraviolet light **(B)**. Circles indicate the infiltrated area on the leaf panels. The fraction in brackets represents the number of HR over the total number of infiltrated leaves.

Since RipTPS_G_ and RipTPS_C_ differs in only three amino acid residues, it’s reasonable to speculate that these three residues are important for the recognition of RipTPS_G_ in *N. tabacum*. The three residues are D^23^, S^290^ and A^483^ in RipTPS_G_. The corresponding three residues in RipTPS_C_ are G^23^, R^290^ and V^483^, respectively (**Figure 1C**). To evaluate the involvement of the three polymorphic amino acid sites in RipTPS_G_ avirulence activity, we generated a series of RipTPS_G_ mutants, including all three single-residue mutants and all three double-residue mutants. In these mutants, we mutated the residues in RipTPS_G_ to the corresponding ones in RipTPS_C_. RipTPS_C_ can be considered as the three-residue mutant of RipTPS_G_. After transiently expressing these mutated genes, we found that all the single-residue and double-residue mutants still elicited strong HR (**Figure 4**). Only when all three residues were mutated (RipTPS_C_), RipTPS_G_-elicited HR was abolished. These results indicate that the three amino acid polymorphisms jointly determine the recognition of RipTPS_G_ in *N. tabacum*.

### Both RipTPS_G_ and RipTPS_C_ suppress flg22-induced ROS burst in *N. benthamiana*

**Figure 5:**
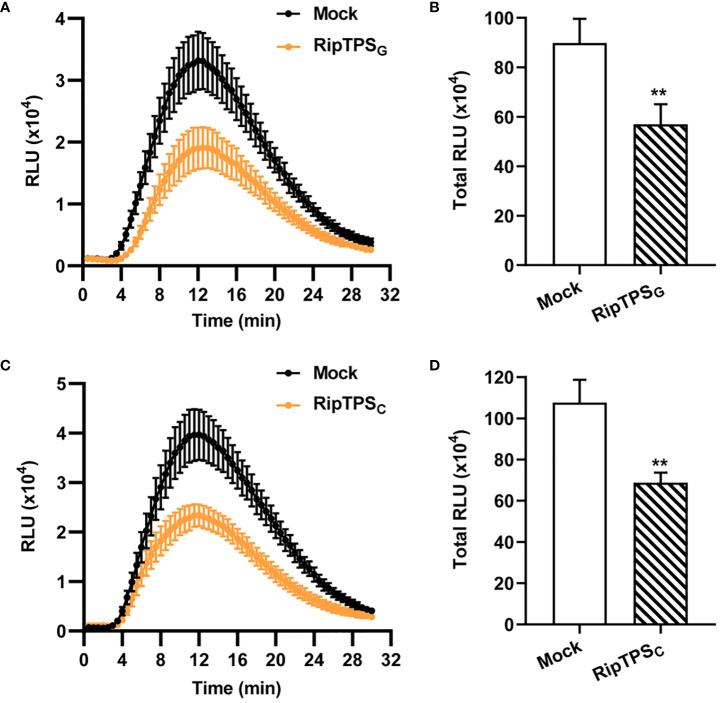
Expression of *RipTPS*_G_ or *RipTPS*_C_ suppresses flg22-induced reactive oxygen species (ROS) burst in *N. benthamiana*. **(A, C)** Time course curve of ROS production in leaves of *N. benthamiana* transiently expressing *LTI6b* (Mock, Control), *RipTPS*_G_ **(A)** or *RipTPS*_C_ **(C)**. Two days after infiltration, leaf discs were treated with 100 nM flg22 elicitor, and ROS production was measured as photon counts for 30 min. **(B, D)** Total ROS accumulation during 30 min in *N. benthamiana* leaves of **(A, C)**, respectively. RLU: relative luminescence units. Data are means ± SE from 14 independent leaf discs. ** indicates significant difference at *p* ≤ 0.01.

*R. solanacearum* usually secretes effectors to interfere with host immunity and promote its infection. To investigate whether RipTPS_G_ affects plant basal defense, we expressed *RipTPS*_G_ in *N. benthamiana* and measured ROS production induced by the bacterial PAMP flg22, which is a major epitope peptide of bacterial flagellin. We found that a peak value greater than 3.3×10^4^ relative luminescence units (RLU) was reached in control plants (Mock, **Figure 5A**). However, in *RipTPS*_G_-expressing plants, the peak value was less than 2.0×10^4^ RLU. Compared with the LTI6b control, *RipTPS*_G_ expression significantly inhibited the total ROS accumulation during a 30 min period (**Figure 5B**). These results suggest that RipTPS_G_ interferes with PAMP-triggered ROS accumulation. Similar effect was also observed for RipTPS_C_ (**Figures 5C, D**), indicating that the three amino acid polymorphisms do not affect its function in suppression of basal defense response.

### RipTPS’s role of suppressing flg22-induced ROS burst in *N. benthamiana* is independent of its TPS enzymatic activity

**Figure 6:**
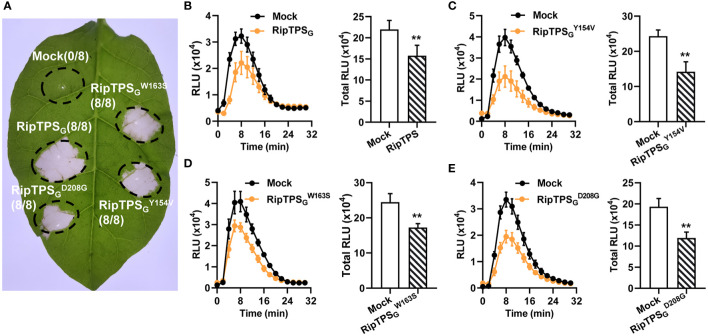
TPS activity is required for neither RipTPS_G_-elicited HR in *N. tabacum* nor suppression of flg22-induced ROS burst in *N. benthamiana*. **(A)** RipTPS_G_-elicited HR in *N. tabacum* is independent of its TPS activity. Leaves of *N. tabacum* were infiltrated with GV3101 carrying *LTI6b* (Mock), *RipTPS*_G_, or *RipTPS*_G_ mutants compromised in its enzymatic activity (RipTPS_G_^Y154V^, RipTPS_G_^W163s^ and RipTPS_G_^D208G^). Photographs were taken 24 hours after infiltration. Circles indicate the infiltrated area on the leaf panels. The fraction in brackets represents the number of HR over the total number of infiltrated leaves. **(B–E)** Suppression of flg22-induced ROS burst by RipTPS_G_ in *N. benthamiana* is independent of TPS activity. Both of time course curve of ROS production and total ROS accumulation in leaves of *N. benthamiana* transiently expressing *LTI6b* (Mock, Control), RipTPS_G_, or RipTPS_G_ mutants were showed. Two days after infiltration, leaf discs were treated with 100 nM flg22, and ROS production was measured as photon counts for 30 min. RLU: relative luminescence units. Data are means ± SE from 14 independent leaf discs. ** indicates significant difference at *p* ≤ 0.01.

Poueymiro et al. (2014) revealed that RipTPS_G_ directs the production of plant signal metabolite trehalose-6-phosphate. They identified three residues essential for its trehalose-6-synthase enzymatic activity and showed that enzymatic activity is not required for RipTPS-elicited HR in *N. tabacum*. Here, we also showed that the three catalytic mutants of RipTPS_G_ (RipTPS_G_^Y154V^, RipTPS_G_^W163S^ and RipTPS_G_^D208G^) elicited HR in *N. tabacum* as well as RipTPS_G_ (**Figure 6A**), confirming the finding of Poueymiro et al. (2014). Since trehalose-6-phosphate is an essential signal molecule in plants, we wonder whether RipTPS’s TPS activity plays a role in its suppression of plant basal defense. We compared the effect of RipTPS_G_ and its three catalytic mutants on flg22-induced ROS burst in *N. benthamiana* and found that all three mutants showed the similar activity to RipTPS_G_ (**Figures 6B** **–E**). This result indicates that the trehalose-6-synthase enzymatic activity is not required for RipTPS’s function in suppression of PAMP-triggered ROS production.

### Deletion of *RipTPS*_G_ does not affect GMI1000-induced HR in *N. tabacum*

**Figure 7:**
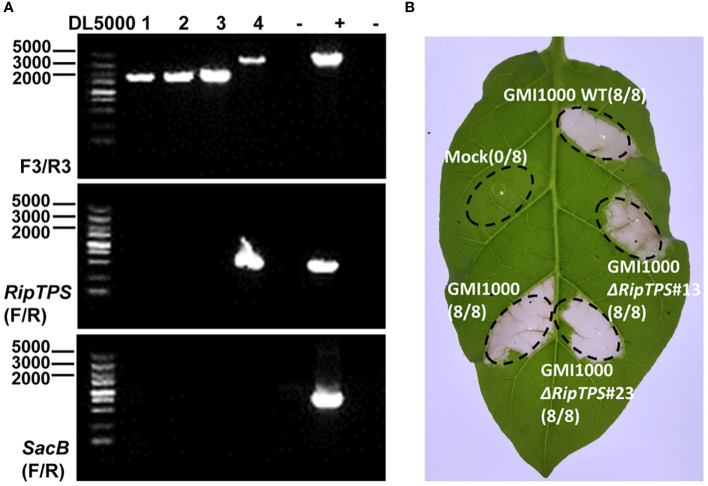
Deletion of *RipTPS* in GMI1000 does not affect its HR induction phenotype in *N. tabacum*. **(A)** Identification of *RipTPS* deletion mutants. The primer pairs F3/R3, RipTPS(F/R) and SacB(F/R) were used for confirmation of double exchange. After double exchange, the *RipTPS* gene was deleted. DL5000: DNA ladder. **(B)** HR induced by wild-type GMI1000 and *RipTPS* deletion mutants. Wild-type GMI1000 or *RipTPS* deletion mutant cells with an OD_600_ of 0.1 were infiltrated into 40-day-old *N. tabacum* leaves, respectively. Photographs were taken 48 hours after inoculation. Water was used as mock. Circles indicate the infiltrated area on the leaf panels. The fraction in brackets represents the number of HR over the total number of infiltrated leaves. GMI1000*ΔRipTPS*#13 and GMI1000*ΔRipTPS*#23 are two independent *RipTPS* deletion mutants.

To further reveal the role of RipTPS in GMI1000-induced HR in *N. tabacum*, we deleted RipTPS in GMI1000 (**Figure 7A**). The RipTPS deletion mutants were infiltrated into *N. tabacum* leaves, and the HR induction was observed. The result demonstrated that two independent *RipTPS* deletion mutants still induced strong HR, which is similar to the wild-type GMI1000 (**Figure 7B**), indicating deletion of RipTPS alone is not sufficient to abolish GMI1000-induced HR in *N. tabacum*.

## Discussion

### RipTPS_G_ acts as a host-specificity avirulence factor

*R. solanacearum* is an aggressive pathogen with a large repertoire of T3Es (Genin and Denny, 2012). Functional characterization of these effectors is critical for understanding the mechanisms of host specificity and pathogenicity in these economically important pathogens (Kim et al., 2021; Yang et al., 2022). However, to date, only few *R. solanacearum* T3Es were characterized for their roles in interaction between *R. solanacearum* and its host/nonhost plants. The model *R. solanacearum* strain GMI1000 cannot infect either *N. benthamiana* or *N. tabacum*, but its close relative CQPS-1 infects both *N. benthamiana* and *N. tabacum*. This feature makes these two *R. solanacearun* strains good models to study host-*R. solanacearum* recognition. We analyzed HR induced by *R. solanacearum* T3Es in *N. tabacum* and *N. benthamiana*, and identified RipTPS_G_ which induced HR in *N. tabacum* but not in *N. benthamiana*, indicating that RipTPS_G_ is recognized in *N. tabacum*. This result was supported by a previous report (Poueymiro et al., 2014).

To further demonstrate whether RipTPS_G_ is an avirulence determinant, we took advantage of the *R. solanacearum* CQPS-1 whose genome has also been sequenced. In contrast to GMI1000, CQPS-1 contains conserved T3SS gene clusters, but is a virulent *R. solanacearum* strain on tobacco (Liu et al., 2017). In the present study, we found that there are only three amino acid polymorphisms between RipTPS proteins of the two strains (**Figure 1**). We then took advantage of this natural variation and obtained the following evidence: (1) RipTPS_G_ elicited HR on *N. tabacum*, but RipTPS_C_ cannot (**Figure 1**); (2) RipTPS_G_ dramatically enhanced expression of HR-related genes *NtHIN1* and *NtHsr203J* in *N. tabacum* (Takahashi et al., 2004), which was significantly compromised when RipTPS_G_ was replaced by RipTPS_C_ (**Figure 2**); (3) The *RipTPS*_G_ expression reduced virulence of CQPS-1 on *N. tabacum* (**Figure 3**). These results demonstrate that RipTPS_G_ is an avirulence determinant that triggers defense in *N. tabacum*, while RipTPS_C_ acts as a virulence T3E. However, expression of *RipTPS*_C_ still slightly enhanced expression of the HR marker genes (**Figure 2**). Similar weak responses of *N. benthamiana* to the virulent alleles has also been reported previously, especially in the overexpression assays (Westerink et al., 2004; Bos et al., 2006).

Compared with other bacterial plant pathogens, *R. solanacearum* has a large repertoire of secreted effectors (Yu et al., 2020), and some of these effectors are functionally redundant (Cunnac et al., 2004; Macho et al., 2010) and balanced for a successful infection (Sang et al., 2020). Deleting RipTPS alone in GMI1000 did not significantly affect *R. solanacearum*-induced HR in *N. tatacum* (**Figure 7**), indicating other avirulence genes exist in GMI1000. Indeed, AvrA (Poueymiro et al., 2009) and RipAW (Niu et al., 2022) from GMI1000 have been reported to act as avirulence factors in *N. tabacum*. Differently, AvrA and RipAW are recognized by both *N. benthamiana* and *N. tabacum* (Poueymiro et al., 2009; Niu et al., 2022). Therefore, RipTPS acts as an avirulence determinant specifically recognized in *N. tabacum*.

### The three amino acid polymorphisms determine the recognition ofRipTPS_G_ in *N. tabacum*

During the coevolution of phytopathogens and plants, pathogens must overcome ETI for further infection through evolution of pathogen effectors that escape or suppress ETI (Jones and Dangl, 2006). Sang et al. (2020) showed that *R. solanacearum* effector RipAY directly inhibited another effector RipE1-triggered ETI in *N. benthamiana* to counteract its perception by plant immune system. Many plant pathogens have also developed strategies to escape host recognition by point mutations, gene deletions, transposon insertions or deliberate mistranslation (Westerink et al., 2004; Vargas-Rodriguez et al., 2021). Escaping recognition by the strategy of point mutations of avirulence factor has been reported in several phytopathogens such as *Cladosporium fulvum* (Westerink et al., 2004) and *Phytophthora infestans* (Bos et al., 2006). Here, only three polymorphic amino acid residues are present in RipTPS between avirulent strain GMI1000 and virulent strain CQPS-1. Site-directed mutagenesis analyses showed that neither the single nor the double amino acid substitution abolished RipTPS_G_-elicted HR. RipTPS_G_-triggered HR was abolished only when all its three polymorphic residues were substituted into the corresponding residues in RipTPS_C_ (**Figure 4**). This finding suggests that all three amino acid residues are required for the recognition of RipTPS_G_ in *N. tabacum*, and RipTPS_C_ evades the recognition through simultaneously mutating these three residues. Poueymiro et al. (2014) pointed out that the C-terminal half (amino acid 336-557) of RipTPS_G_ alone could trigger the HR-like response. Our result indicated that the N-terminal region of RipTPS also contributes to its recognition in *N. tabacum*, since both D23 and S290 residues are required for RipTPS_G_-induced HR in our result (**Figure 4**). These three residues may affect three-dimensional structure of RipTPS protein or interactions between RipTPS and other plant proteins.

### RipTPS exhibited defense suppression activity in *N. benthamiana*

ROS play a crucial role in biotic stress sensing and activation of stress-response networks (Mittler et al., 2022). ROS burst is a hallmark event for plant basal defense (Segonzac et al., 2011; Nakano and Mukaihara, 2019a). Here, we showed that expression of *RipTPS*_G_ suppressed flg22-induced ROS burst in *N. benthamiana* (**Figure 5**), indicating that RipTPS_G_ interferes with plant immune response in *N. benthamaina*. Similar phenomenon was also observed in other T3Es, such as PopP2 and AvrA which are known as avirulence determinants but contribute to pathogen virulence in susceptible host (Macho et al., 2010). Interestingly, expression of *RipTPS*_C_ also suppressed flg22-induced ROS burst in *N. benthamiana* (**Figure 5**), indicating that RipTPS_C_ retains its ability to suppress plant defense response and the three amino acid polymorphisms do not affect RipTPS’s virulence activity.

### TPS activity is involved in neither RipTPS_G_’s avirulence nor virulence functions

The most striking feature of RipTPS is its inherently trehalose-6-synthase enzymatic activity as its name suggests (Poueymiro et al., 2014). Trehalose, a non-reducing sugar, has been found in multiple microbes ranging from bacteria to yeast and in plants. In plants, trehalose not only serves as a reserve carbohydrate and structural components of cells but also acts as a signaling molecule, and it plays important physiological roles in plant growth and stress resistance (Wahl et al., 2013; Sarkar and Sadhukhan, 2022; Shao et al., 2022). Interestingly, in some phytopathogens, trehalose metabolism has emerged as an essential player in virulence-associated phenotypes and proficient initial plant infection (Tournu et al., 2013). For instance, trehalose biosynthesis promotes *Pseudomonas aeruginosa* virulence in plants by promoting the acquisition of nitrogen-containing nutrients (Djonovic et al., 2013). However, we demonstrated that the TPS enzymatic activity was not required for its avirulence function, which is consistent with the result of Poueymiro et al. (2014). We also showed that the TPS enzymatic activity was not required for its function in suppression of PAMP-induced ROS production (**Figure 6**). Further studies are needed to elucidate the role of the TPS activity of RipTPS in *R. solanacearum*-plant interactions.

In summary, in this study we demonstrated that RipTPS from *R. solanacearum* strain GMI1000 is an avirulence determinant recognized in *N. tabacum*, whereas the RipTPS homolog from another strain CQPS-1 can escape this recognition. The three amino acid residues of RipTPS are jointly required for the recognition of RipTPS_G_ in *N. tabacum*. Both RipTPS_G_ and RipTPS_C_ retain their ability to suppress plant defense responses. The TPS activity of RipTPS is not required for its avirulence function and also virulence activity of suppressing PAMP-induced ROS burst. It will be a promising direction to identify the corresponding resistance gene recognizing RipTPS_G_ in *N. tabacum* and also to elucidate the role of TPS activity of RipTPS in *R. solanacearum*-plant interactions in the future.

## Data availability statement

The original contributions presented in the study are included in the article/**Supplementary Material**. Further inquiries can be directed to the corresponding author.

## Author contributions

YA and MZ conceived and designed the experiments. JC, ZX, XO, and PC performed the experiments. YA, RW, PL and MZ analyzed the data. YA, JC and MZ drafted and modified the manuscript. All authors contributed to the article and approved the submitted version.

## Funding

This research was supported by the National Natural Science Foundation of China (32072399, 32272641), the Fundamental Research Funds for the Central Universities (GK202201017), the Program of Fujian Key Laboratory for Monitoring and Integrated Management of Crop Pests (MIMCP-202203), and the Joint Research Project of FAAS (DWHZ2021-13).

## Conflict of interest

The authors declare that the research was conducted in the absence of any commercial or financial relationships that could be construed as a potential conflict of interest.

## Publisher’s note

All claims expressed in this article are solely those of the authors and do not necessarily represent those of their affiliated organizations, or those of the publisher, the editors and the reviewers. Any product that may be evaluated in this article, or claim that may be made by its manufacturer, is not guaranteed or endorsed by the publisher.
