# Supplementary material for: Three amino acid residues are required for the recognition of Ralstonia solanacearum RipTPS in Nicotiana tabacum
Source: Front Plant Sci. 2022 Oct 13;13:1040826. doi: 10.3389/fpls.2022.1040826 (PMC9606615; doi:10.3389/fpls.2022.1040826)
Supplement: Supplementary file 1 [file Table_1.docx]

Table S1. Primers used in this study

| **Primer name** | **Primer sequence 5' to 3'** |
| --- | --- |
| RipTPS_G_^D23G^-F | CCCGACACGCCCGGTGCCGGCGAAGCCCAACGCGCCGATC |
| RipTPS_G_^D23G^-R | GATCGGCGCGTTGGGCTTCGCCGGCACCGGGCGTGTCGGG |
| RipTPS_G_^S290R^-F | GTCCGAGCGCCGCCATGACCGTGTCCACGCCTTCCCGATC |
| RipTPS_G_^S290R^-R | GATCGGGAAGGCGTGGACACGGTCATGGCGGCGCTCGGAC |
| RipTPS_G_^A483V^-F | CCGGGCCGCCACCGCCGATGTGTACGCGCAGGCCCTGTCCATG |
| RipTPS_G_^A483V^-R | CATGGACAGGGCCTGCGCGTACACATCGGCGGTGGCGGCCCGG |
| RipTPS_G_^Y154V^-F | CCGATTACGAGGGCTACGTGGCGGGGTTCTCG |
| RipTPS_G_^Y154V^-R | CGAGAACCCCGCCACGTAGCCCTCGTAATCGG |
| RipTPS_G_^W163S^-F | CGAATTCGGTGCTCAGCCCCATCTTCCACGAG |
| RipTPS_G_^W163S^-R | CTCGTGGAAGATGGGGCTGAGCACCGAATTCG |
| RipTPS_G_^D208G^-F | CTGTGGGTCCACGGCTACCATCTCATCCCG |
| RipTPS_G_^D208G^-R | CGGGATGAGATGGTAGCCGTGGACCCACAG |
| RipTPS_G_^up^-F1 | gagctcggtacccggggatccGTTGCTGTTGAGCAGTTGGTT |
| RipTPS_G_^up^-R1 | TGCGTATTCGTATTGCCTTTG |
| RipTPS_G_^down^-F2 | CAAAGGCAATACGAATACGCAGCCGGCCGTGCCCGCGCGTCGCAT |
| RipTPS_G_^down^-R2 | tgcctgcaggtcgactctagaTGATAATCGCCCAGCAGTTC |
| RipTPS_G_-F | ATGCGCCCGATTATTTCCAC |
| RipTPS_G_-R | ATTCAAATCCGCCCATTTCAC |
| RipTPS_G_-F3 | TCAGGTACTGCGGAAAGTGG |
| RipTPS_G_-R3 | CGCCAGGTTGTTTTTCTTCT |
| SacB-F | AGGCCTGGACGTTTGGGACA |
| SacB-R | TCCGCGGGAGTCAGTGAAC |
| NtHIN1-F | AGCCTATTATGGCCCTTCC ATT |
| NtHIN1-R | TGGCTCAACGATTACGCA |
| NtHsr203J-F | GCACGAAACCTGGATGG |
| NtHsr203J-R | GGCCGTGGCGGTGATA |
| NtEF1α-F | GAGATGGGGACAAAGGGGAT |
| NtEF1α-R | TGCTGCTGTAACAAGATGGATGC |
